# EANM position paper on challenges and opportunities of full-ring 360° CZT bone imaging: it’s time to let go of planar whole-body bone imaging

**DOI:** 10.1007/s00259-024-06906-4

**Published:** 2024-09-11

**Authors:** Richard Graham, David Morland, Sarah Cade, Laetitia Imbert, Emmanouil Panagiotidis, Jens Kurth, Frédéric Paycha, Tim Van den Wyngaert

**Affiliations:** 1 Department of Radiology, Royal United Hopital, Bath, UK; 2 Department of Nuclear Medicine, Institut Jean-Godinot, Reims, France; 3 Department of Medical Physics, Royal United Hopital, Bath, UK; 4 Department of Nuclear Medicine and Nancyclotep Imaging Platform, CHRU Nancy, Université de Lorraine, IADI, INSERM U1254, Nancy, France; 5 PET/CT Oncology Center ’Theageneio’, Thessaloniki, Greece; 6 Department of Nuclear Medicine, Rostock University Medical Center, Rostock, Germany; 7 Department of Nuclear Medicine, Hôpital Lariboisière, Assistance Publique-Hôpitaux de Paris, Paris, France; 8 Department of Nuclear Medicine, Antwerp University Hospital, Edegem, Belgium; 9 Faculty of Medicine and Health Sciences (MICA – IPPON), University of Antwerp, Wilrijk, Belgium

**Keywords:** Bone scintigraphy, Ring geometry, Cadmium-Zinc-Telluride, CZT, 360° SPECT/CT

## Abstract

The introduction of smaller footprint, more sensitive Cadmium-Zinc-Telluride (CZT)-based detectors with improved spatial and energy resolution has enabled the design of innovative full-ring 360° CZT SPECT/CT systems (e.g., VERITON^®^ and StarGuide™). With this transformative technology now aiming to become mainstream in clinical practice, several critical questions need to be addressed. This EANM position paper provides practical recommendations on how to use these devices for routine bone SPECT/CT studies, facilitating the transition from traditional planar whole-body imaging and conventional SPECT/CT to these novel systems. In particular, initial guidance is provided on imaging acquisition and reporting workflows, image reconstruction, and CT acquisition parameters. Given the emerging nature of this technology, the available evidence base is still limited, and the proposed adaptations in workflows and scan protocols will likely evolve before being integrated into definitive guidelines. In the meantime, this EANM position paper serves as a comprehensive guide for integrating these advanced hybrid SPECT/CT imaging systems into clinical practice and outlining areas for further study.

## Introduction

Over the last few years, Cadmium-Zinc-Telluride (CZT)-based digital solid-state SPECT technology has become widely available, providing excellent count sensitivity, spatial resolution, and energy resolution, and enabling significant reductions in administered activities or acquisition time. While CZT crystals are not a prerequisite for high-resolution imaging and have intrinsic detector efficiencies similar to traditional systems, they have proved crucial in designing smaller footprints and lighter-weight detectors. These compact detectors, combined with advances in collimator design and geometries, have served as enabling technologies to develop innovative full-ring 360° detector layouts, surpassing the classic design of the dual-head Anger gamma camera [1]. This new architecture features CZT detectors and a fixed tungsten-based collimation system. In combination, the detectors can move independently in the axial plane and come very close to the patient, improving sensitivity, energy resolution and image contrast [2]. Two systems with this design are currently available: the VERITON^®^ [4] from Spectrum Dynamics and the StarGuide™ from GE HealthCare [3]. With about 100 installations worldwide, our imaging community has now reached a tipping point with this technology aiming to become mainstream and impact patient management, similar to how the introduction of dedicated cardiac CZT devices improved nuclear cardiology practice [5]. This new technology raises many questions on how it should be used in routine clinical practice, including how to adapt scan protocols. More fundamental issues also need to be addressed: are these devices true general-purpose gamma camera alternatives or tailored primarily for theranostics and dosimetry applications?

At present, bone imaging with ^99m^Tc-labelled bisphosphonates comprises approximately 25–35% of SPECT indications [6–9]. It has greatly benefited from the introduction of hybrid SPECT/CT, particularly in the post-operative setting. The current EANM guidelines for bone scintigraphy date from before the development of full-ring 360° CZT SPECT/CT systems, and some recommendations may need to be reviewed when implementing these new scanners [10]. Indeed, bone scan has been performed for decades on a planar imaging basis only. Since the patient’s radiation exposure is solely related to the administered radiopharmaceutical activity, the vast majority of bone scan protocols are not limited to the region of interest but include a whole-body planar acquisition.

The arrival of SPECT/CT gamma cameras 20 years ago changed this paradigm, with SPECT/CT acquisitions considered an add-on to planar imaging [11]. Although the sensitivity and specificity of SPECT/CT are higher than planar imaging, the incremental exposure due to the CT - depending on the acquisition settings and scan length - has led to a rationalisation of the use of SPECT/CT. Historical tough-to-let-go habits and exposure optimisation led departments to perform whole-body planar imaging first and then define anatomical segments for SPECT/CT add-on acquisitions. However, this reasoning no longer applies to full-ring 360° CZT SPECT/CTs; another paradigm shift is necessary.

At this time, it is too early to compose evidence-based guidelines for the use of full-ring 360° CZT SPECT/CT devices, as not enough data has been published on their use. Only a handful of publications are currently available, especially detailing their use for bone imaging [12, 13]. Therefore, we present initial expert opinion in this EANM position paper as an adjunct to the current EANM practice guideline on bone scintigraphy on how workflows and scan protocols can be adjusted for these novel devices. In addition, we discuss how this technology can be used for patient benefit beyond theranostics, with a focus on bone applications.

## Full-ring 360° CZT SPECT/CT systems

### System architecture

Full-ring 360° CZT SPECT/CT systems are equipped with detectors that swivel during the scan acquisition, and the 360-degree gantry design optimises three-dimensional imaging with the digital detectors being as close to the patient as possible. The StarGuide™ system uses rapid contouring of the patient at the start of the scan to already have the detectors in the correct position for each bed position to reduce scan time. The VERITON^®^ uses capacitance-based sensors in the heads to position them immediately in the correct position. The spatial resolution in both systems is below 5 mm. The StarGuide™ has an energy range of up to 270 keV, whereas three versions of the VERITON^®^ CT are available with energy ranges of up to 200, 300 and 400 keV, respectively. This is mainly relevant for imaging ^131^I (360 keV) and both photopeaks of ^177^Lu (113 keV and 208 keV).

Both systems have modern diagnostic multi-detector CTs, with 16 or 64-slice options available, which can be optimised from ultra-low dose for attenuation correction only to full diagnostic image quality. There is no significant advantage for bone imaging indications of 64 over 16 slices. Both vendors utilise metal artefact reduction. These SPECT/CT systems include state-of-the-art radiation protection measures, such as adequate beam filtering and iterative reconstruction, which reduce the additional radiation exposure from the CT.

### Quality control

The majority of acceptance testing procedures for the SPECT portion of the device that are routinely performed on conventional dual-head systems cannot be performed on the full-ring 360° CZT SPECT/CT systems. This is mainly because no planar images can be acquired, and the collimators cannot be removed from the individual detectors. However, custom QC tools specifically designed for these systems are supplied by the vendors. However, this may have implications for current harmonisation efforts in SPECT/CT, and more work is needed to provide a transparent and consistent framework across vendors [14]. There is no question that regular quality control of the CT component in accordance with manufacturer recommendations and local requirements must be an indispensable part of the operator’s quality control program.

## Implications for bone SPECT/CT imaging

### Comparison with traditional SPECT/CT cameras

On full-ring 360° CZT SPECT/CT systems, a SPECT/CT acquisition of the trunk (skull base to mid-thighs) field of view (FOV) can be performed in about 10 min, and a whole-body FOV (vertex-to-toes) in about 22 min acquisition time. On a traditional SPECT/CT, it takes about 20–30 min of acquisition time to perform a trunk SPECT/CT and about 45 min to perform a whole-body study. The acquisition time per bed position can be adjusted according to the scanned anatomic region. It is also possible to perform a whole body SPECT on a 360° CZT system with a coregistered CT limited to a part of the body. However, omitting the CT will result in a loss of attenuation correction of the SPECT reconstruction. Therefore, the acquisition of SPECT/CT is generally the preferred workflow on these systems. When scanning small regions of interest (e.g., spine, brain, cardiac, thyroid), full-ring 360° CZT systems can be used in focused mode to increase sensitivity further, with the swivel of the detectors limited to the area of interest.

### Image acquisition

The transition from traditional whole-body and SPECT/CT cameras to advanced full-ring 360° CZT SPECT/CT devices marks a significant evolution, offering significant advancements and opportunities in bone scintigraphy. These include enhanced imaging capabilities with improved spatial resolution, faster acquisition times, and the ability to perform simultaneous multi-angle projections. Furthermore, these systems provide more flexible and optimized acquisition protocols, reducing the need for high injected activity and lowering radiation exposure. They also simplify patient positioning and improve comfort by shortening scan durations. These issues, along with their implications for clinical practice and patient care, will be discussed in more detail below.

### Image reconstruction

All images are acquired natively in tomographic mode on full-ring 360° CZT SPECT/CT systems. For the SPECT part, resolution recovery and iterative reconstructions are utilised, resulting in fully quantitative data sets that produce standardised uptake values (SUV). Additionally, proprietary reconstruction algorithms can be used to enhance the images further depending on local preference (e.g., partial volume correction [PVC] and penalized likelihood reconstruction).

Specifically, published SPECT reconstruction settings for bone imaging on the VERITON^®^ system suggest using the OSEM algorithm with 4 iterations and 8 subsets with attenuation, scatter, and partial volume correction, and point spread function recovery (kernel inter-iteration filter: 0.2; post-reconstruction median filter: 3 × 3 × 3 voxels) [12, 15]. For the StarGuide™ system, the manufacturer’s block sequence regularisation expectation-maximisation (BSREM) penalized likelihood reconstruction algorithm (Q.Clear™) using the relative difference prior (RDP) method, and 10 iterations and 10 subsets was reported as optimal (gamma: 2.0; beta for non-attenuation corrected images: 0.08; beta for attenuation corrected images: 0.4) with a scan time of 4 min per bed position [13].

### Image display

It is suggested that the following views are used: 3-plane orthogonal native SPECT with attenuation correction (AC), CT with metal artefact reduction (if metal is present) and fused SPECT/CT images. Native maximum intensity projection (MIP) images and fused CT MIPs are of particular benefit to demonstrate pathology to referrers and for referrers to discuss findings with patients. In addition, standardising the display scale may also provide a better reference of the intensity of uptake and facilitate longitudinal comparisons. It is recommended to standardise the display scale of SUVs using a fixed SUV_max_. While personal preference may determine this scale, a recommended range is from 0 to between 10 and 15 SUV_max_. This practice is similar to [^18^F]NaF-PET/CT and [^18^F]-FDG-PET/CT reporting and familiar to most nuclear medicine physicians, with most centres using a 0–10 SUV_max_ display scale [16]. The use of SUV can facilitate longitudinal measurement between scans to potentially aid treatment response assessment [17]. While reference values have been published for some conditions, more work needs to be undertaken to determine if quantification using SUV is a useful parameter in the interpretation of bone SPECT/CT [18–20]. In addition, volumetric SUV-based SPECT/CT metrics of bone remodeling (analogous to Fluoride Tumor Volume [FTV] on [^18^F]NaF-PET/CT) or combined with mean lesion uptake (comparable with Total Lesion Fluoride [TLF] uptake on [^18^F]NaF-PET/CT) should be further investigated [21].

### Reporting strategy

**Fig. 1:**
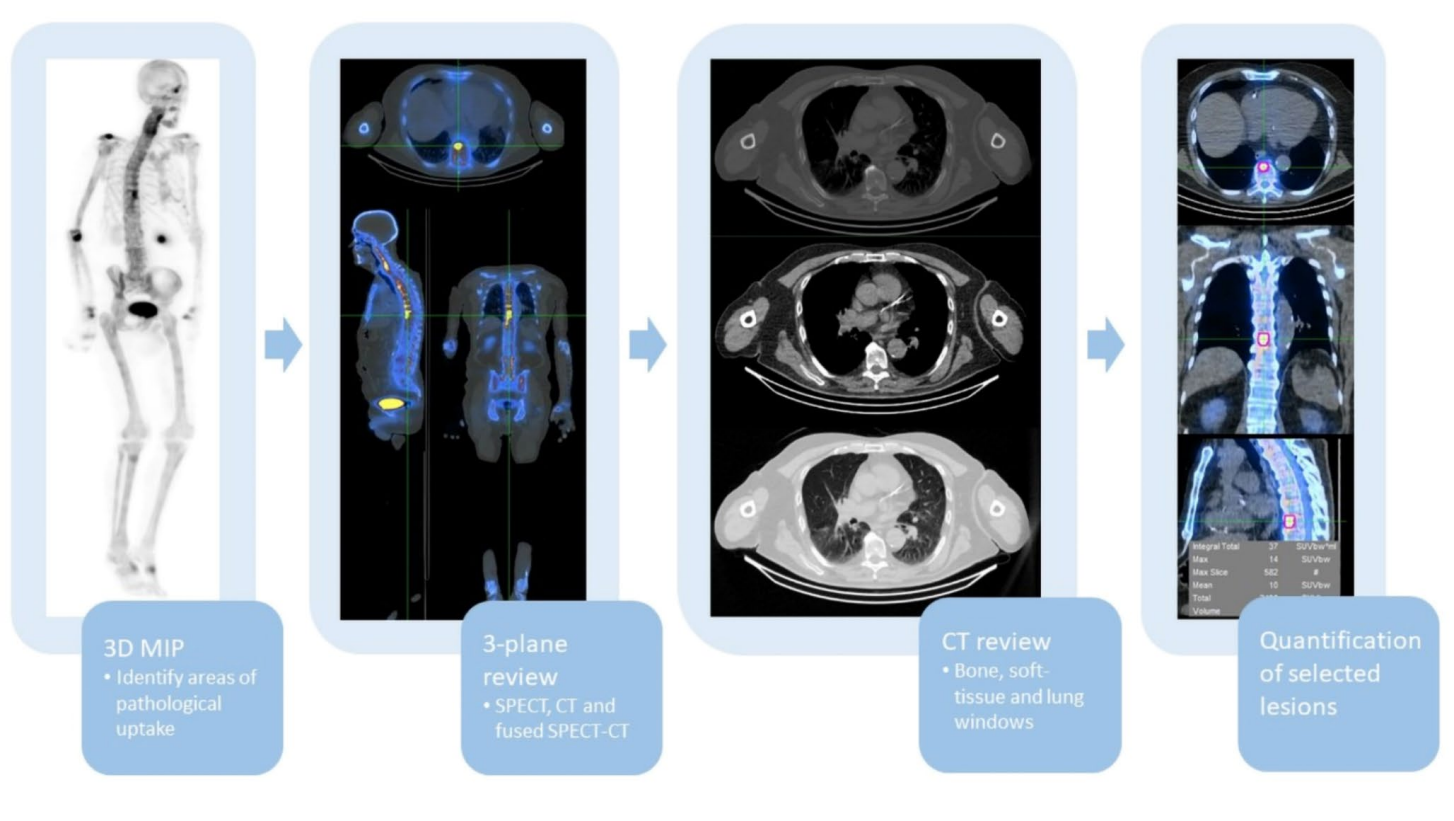
Suggested SPECT/CT reporting workflow

Initially, reviewing the rotating 3D MIP will help identify the areas of pathologically increased tracer uptake. Next, careful cross-referencing with the 3-plane SPECT, CT and fused SPECT/CT images is advised. Then, a review of the CT dataset on bone, soft tissue and lung windows should be performed to look for incidental findings. Quantification of uptake using SUV_max_ or other SUV-based metrics can help classify focal sites of uptake as pathologically increased (Fig. 1), yet further studies are needed to determine appropriate reference ranges. Adhering to a fixed reading and reporting workflow can help overcome the challenge of effectively dealing with the large datasets generated when performing whole-body SPECT/CT studies, which will take more time and effort to interpret. Given the large datasets created with whole-body SPECT/CT acquisitions, an increase in reporting times is indeed expected, with complex studies requiring up to 30–40 min, depending on experience and indication. In addition, the use of reporting templates may help to streamline the reporting process.

### Letting go of planar

The EANM practice guideline for bone scintigraphy states that planar whole-body images are routinely acquired and that sequential planar acquisitions are used in assessing various diseases [22]. According to the same document, multimodality SPECT/CT imaging is subsequently indicated for assessing lesions that are equivocal on planar bone scintigraphy or localised pain syndromes with normal findings on planar scintigraphy. Obviously, these statements require revision with the introduction of full-ring 360° CZT SPECT/CT systems. Previously the lack of planar images has been perceived as a barrier in reporting whole-body SPECT/CT studies, as these often serve as a reference for the intensity of tracer uptake.

**Fig. 2:**
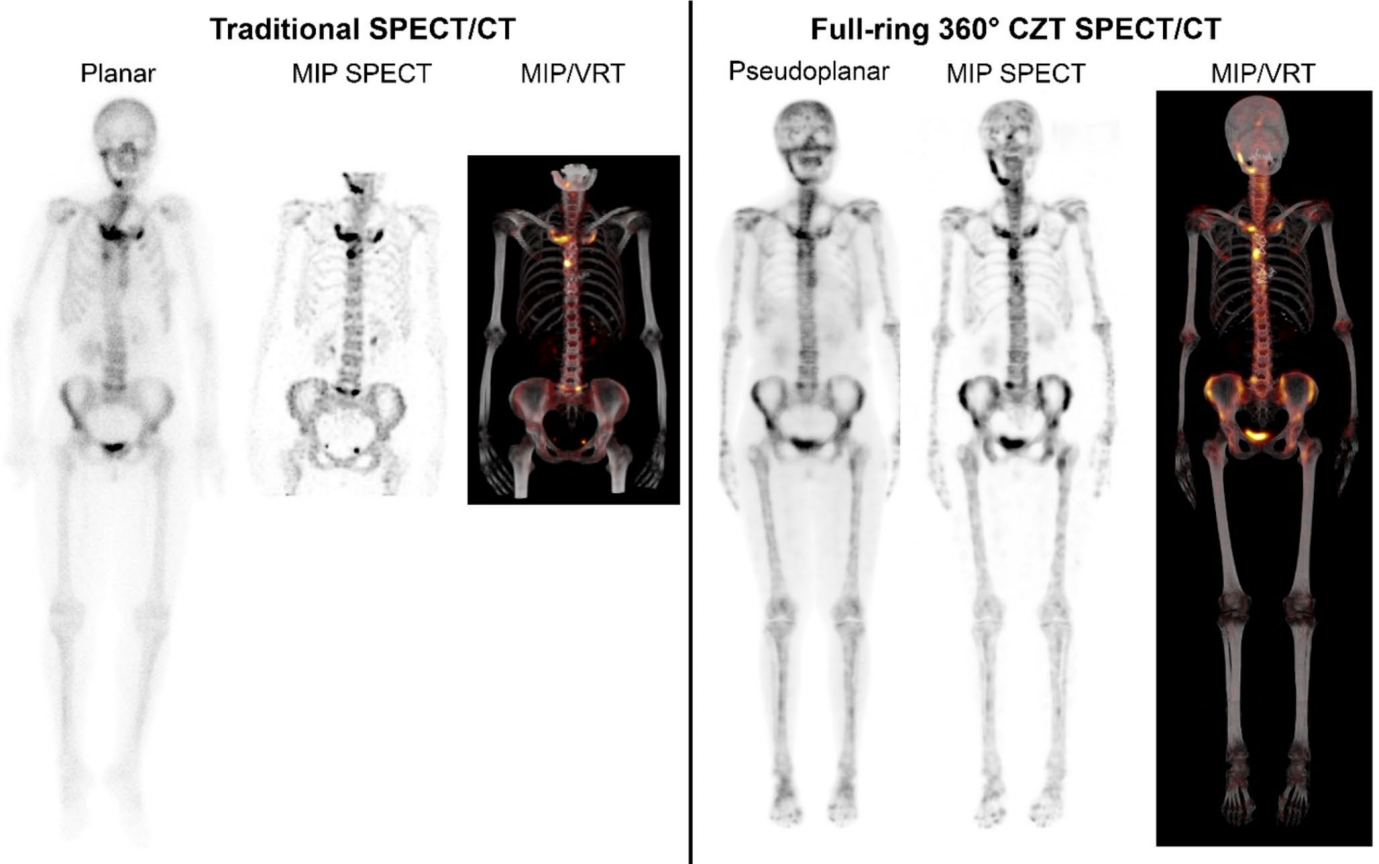
Comparison of traditional anterior planar whole-body view and reconstructed pseudoplanar using full-ring 360° CZT SPECT/CT and respective fused Volume Rendering Technique (VRT) image in the same patient with synovitis, acne, pustulosis, hyperostosis, osteitis (SAPHO) syndrome

In order to facilitate this transition, several alternatives can be suggested for producing pseudo-planar reconstructions based on the SPECT acquisition: maximum intensity projections (or summed coronal slices) and retroprojected images [23–25]. This involves recreating a projection image from the reconstructed volume (Fig. 2). However, one should wonder if holding on to planar images is even useful in the first place, with SPECT/CT becoming the standard image acquisition mode. A learning curve in the interpretation of whole-body bone SPECT/CT studies acquired on a full-ring 360° CZT camera is normal when transitioning from planar imaging, similar to that seen in readers starting with [^18^F]-NaF-PET/CT for oncological staging [26]. Therefore, we do not recommend using pseudo-planar images when reporting SPECT/CT studies indefinitely, except as a temporary aid to facilitate transitioning from planar to SPECT/CT reporting. Some centres have used SPECT/CT only without pseudo-planar images. In this situation, they advocate the use of fused spinning MIPs, which obviate the need for pseudo-planar images for the referring clinicians to review. Importantly, referring clinicians may require support in understanding the new study layout and screen captures appearing in picture archiving and communication systems (PACS), including key images, to avoid misinterpretation of the results.

## Bone SPECT/CT in malignant disease

### How to define the CT acquisition parameters?

When using a full-ring 360° CZT camera, the CT is used for attenuation correction, and it is recommended that all bone imaging is performed with CT to obtain this benefit. However, the capabilities of the integrated CT scanners allow for diagnostic image quality at the expense of higher patient exposure. While iterative reconstruction techniques for CT are now widely available and can significantly reduce radiation doses without loss of image quality, the hybrid imaging community has struggled with defining the preferred CT acquisition parameters used in hybrid SPECT/CT studies to balance image quality and dose. This has resulted in the misleading nomenclature of low and high-dose CT acquisitions and large practice variations [1, 27, 28]. Critically, it needs to be emphasised that different CT protocols should be created as part of the SPECT/CT workflow depending on the indication of the study (e.g., which disease and clinical question) and the purpose of the CT acquisition (e.g., attenuation correction, localisation, optimal diagnostic quality), rather than on the body region alone. Subsequently, every CT acquisition protocol needs to be checked regularly and optimised regarding compliance with locally applicable dose reference levels (DRL) [29].

Recognising the different and unique roles of CT in the setting of integrated hybrid imaging, a working definition is proposed using the term auxiliary CT, describing CT acquisitions that are not optimised for maximum diagnostic yield, in contrast to those performed as a stand-alone diagnostic CT of the same region [30]. Future work should focus on more accurately defining the term auxiliary CT within the context of different bone SPECT/CT indications and identifying appropriate DRLs.

When performing a typical oncological staging whole-body study, a CT scan for attenuation correction and localisation purposes is suggested, resulting in a dose of approximately 3 mSv. In comparison, a CT optimised for attenuation correction only would yield an effective dose of 0.3 mSv, whereas for a diagnostic study of the whole-body will result in an effective dose of up to 14 mSv.

### Can acquisitions of the trunk replace routine whole-body imaging?

The advantages of a whole-body acquisition for oncological indications are the full knowledge of the extent of the skeletal involvement and potentially avoiding distal pathological fractures with timely surgical management. In contrast to planar whole-body bone imaging, SPECT/CT bone imaging is usually performed using a two FOV trunk acquisition or a three FOV whole-body study, as the axial FOV on traditional SPECT/CT cameras is limited to approximately 40 cm. The full-ring 360° CZT SPECT/CT systems use bed positions of up to 32 cm on the VERITON^®^ and 27.5 cm on the StarGuide™ to scan subsequent regions of the patient, similar to PET/CT devices. Performing a whole-body over a trunk acquisition adds a time penalty of about 9 min (3 bed positions at 3 min each), which impacts patient comfort and workflow. In addition, there is a limited additional radiation exposure for the patient due to the larger scan range of the CT, even though the extremities are considered to have a lower risk of radiation-induced cancer. In addition, it slightly increases the difficulty of reporting, as whole-body SPECT/CT datasets not only consist of more images but require additional zoom and pan actions to navigate the data. Acquisitions of the trunk are easier to navigate as these are generally about half the craniocaudal dimension.

To balance the disadvantages and benefits, it is important to realise that metastases distal to the elbows and knees (acrometastases) are rare, with an estimated prevalence of 0.1% of all bone metastases, and that historically only 10% of these are reported to occur without centrally located metastases [31, 32]. More recently, in a comparative study of whole-body planar imaging and two FOV bone SPECT/CT, no isolated metastases in the extremities were encountered [33]. Similarly, the initial single-centre experience with a full-ring 360° CZT SPECT/CT system in 117 consecutive oncology patients (mainly with prostate, breast and lung carcinoma) revealed malignancy below the lesser trochanters in 16 (14%) of patients, regardless of metastases elsewhere in the skeleton [34]. Taken together, it is acceptable to limit the SPECT/CT field to the trunk, as done in the mainstay of practice in PET/CT and is deemed sufficient for most oncology indications [35].

### Suggested acquisition workflow

The full-ring 360° CZT SPECT/CT systems offer the possibility of modifying the acquisition time per bed position, enabling optimisation of the study duration while maintaining adequate count statistics in the relevant body areas [12]. An example of acquisition workflow for a typical oncological staging study is presented in Table 1, with corresponding CT acquisition parameters in Table 2. For oncological purposes, the acquisition can be performed 2–3 h after the administration of 8–10 MBq/kg of ^99m^Tc-labelled bisphosphonates (Hydroxydiphosphonate (HDP)/HydroxymethyleneDiphosphonate (HMDP), Methylene Diphosphonate (MDP) or diphosphono propanodicarboxylic acid (DPD)). Patients empty their bladder immediately prior to acquisition. The patient is placed in a supine position with arms by their side. Number of bed positions: 3 min for the head, 2 × 5 min for the chest and abdomen, and 3 × 3 min for the legs, yielding a total scan time of 22 min (excluding patient positioning and table movements). The thighs and legs can be omitted in order to minimise the total scan time to 13 min.

**Table 1:** Suggested SPECT/CT acquisition protocols

Indication	Minimal SPECT field-of-view	CT protocol	Series
Oncology bone staging	*Skull vertex to proximal thigh*: • Head: 1 bed position (3 min) • Body: 2 bed positions (2 × 5 min)	*With delayed phase*: • Localisation or full diagnostic quality CT	• MIP: NM; NM/CT • MPR: NM; CT; NM/CT
Post-operative spinal imaging	*Spine region of interest*: • 1 bed position (10 min) *With pelvis (if lumbar spine)*: • 1 bed position (10 min)	*With delayed phase*: • Localisation or full diagnostic quality CT	• MIP: NM; NM/CT • MPR: NM; CT; NM/CT
Arthroplasty imaging	*Vascular phase*: • 1 bed position (90 s) *Bloodpool phase*: • 1 bed position (5 min; from minute 2 to minute 7) *Delayed phase*: • 1 bed position (8 min)	*With early phase*: • Attenuation correction *With delayed phase*: • Full diagnostic quality CT	*With early phase*: • MPR: NM; ldCT; NM/CT *With delayed phase*: • MIP: NM; NM/CT • Delayed MPR: NM; CT; NM/CT

*Abbreviations* MIP: Maximum intensity projection; MPR: multiplanar reconstruction (axial, coronal and sagittal); NM: nuclear medicine alone; CT: computed X-ray tomography; NM/CT: fused nuclear medicine and CT acquisitions

**Table 2:** Suggested auxiliary CT acquisition parameters depending on the purpose of the CT dataset

	Attenuation correction	Localisation	Full diagnostic quality
**Example parameters**	• 100 kV, CTDI_vol_ = 1.5 mGy • Pitch 1.0	• 120 kV, CTDI_vol_ = 5.5 mGy • Pitch 1.0	• 120 kV, CTDI_vol_ = 35 mGy • Pitch 1.0

The above workflow can be adapted depending on the desired trade-off between a fast scan time or less injected activity. Currently, studies have adhered to the previously recommended activities for ^99m^Tc-labelled bisphosphonates, even though data from whole-body CZT cameras using a conventional design have suggested that further reductions in injected activity should be possible while maintaining the benefit in scan time [10]. However, expert experience shows that shorter acquisition times per bed position may overly depend on the partial volume correction (PVC) methods, which can introduce artefacts when used with noisy data. More work is needed to define the best trade-off in scan time and administered activity in this setting.

## Bone SPECT/CT in benign disease

### How to define the CT acquisition parameters?

Using the framework of optimised auxiliary CT discussed above, it is advised to define multiple SPECT/CT acquisition protocols, tailoring the CT acquisition parameters primarily on the various benign conditions and indications rather than solely on the region or joint (e.g., separate protocols for non-operated joints and after arthroplasty). It is recommended that the use of fully diagnostic quality CT is limited to specific regions of interest in order to limit the additional dose to the patient. However, following this logic, every auxiliary CT protocol will still result in the lowest possible patient exposure with regards to the expected outcome, making the terms low and high-dose CT obsolete. Indeed, doses may be justifiably higher for acquisitions that are optimised for fully diagnostic yield compared to those intended for attenuation correction and localisation only. From this library of protocols, the appropriate SPECT/CT acquisition workflow can then be selected based on the indication of the study and the clinical question (Table 2). Ultimately, integrating CT with SPECT is essential for extracting the maximum diagnostic information, as it enhances image quality and quantification, and promotes the routine use of SPECT/CT to fully leverage the functional and anatomic data, as has been the practice in PET/CT for a long time.

### How to perform perfusion and bloodpool acquisitions?

The vascular phase is traditionally acquired using planar imaging, as technical limitations of SPECT include the problem of reconstructing a volume from projections recorded from different angles while the tracer distribution changes rapidly over time [36, 37]. For the first time, full-ring 360° CZT SPECT/CT systems have overcome this limitation by simultaneously acquiring projections from all angles in list mode [38]. In addition to providing the same information obtained until now by planar dynamic studies, it is anticipated that this new approach will improve the existing methods of quantifying tracer kinetics in bone disease [39]. Overcoming the current clinical limitations of planar vascular phase imaging, this technique will also drive research into novel metrics of disease activity in the skeleton as well as other organs [40–42]. In routine clinical practice, the SPECT technique has largely been ignored for blood pool phase bone imaging, but increasing evidence supports the clinical impact these acquisitions can have on interpretation, particularly in various clinical situations in rheumatology and orthopaedics [43–46].

Depending on the specific full-ring 360° CZT SPECT/CT system, acquiring a multi-phase bone SPECT/CT may include performing more than one CT. In that case, it is recommended to perform an auxiliary CT scan optimised for attenuation correction only for the early-phase imaging to reduce the patient’s overall radiation exposure.

### Suggested acquisition workflow

Whole-body studies can be performed in the same way as described previously for oncological staging studies. While illustrative workflows are presented below for selected typical clinical indications, it is essential to note that these are not exhaustive. Indeed, comprehensive protocols will be necessary to address the wide range of other clinical indications for which bone SPECT/CT is recommended [10]. Still, the individual steps can be used as building blocks for other indications. Additional details are provided in Tables 1 and 2.

#### Post-operative spinal imaging

Administered activity: 8–10 MBq/kg of ^99m^Tc-labelled bisphosphonates (HDP/HMDP, MDP or DPD). Late phase acquisition is performed 2–3 h post tracer injection. Bloodpool imaging with auxiliary CT for attenuation correction purposes is not generally recommended, as the high background activity limits the interpretation of the images. Patients empty their bladder immediately prior to acquisition if it will be within the field of view. The patient is in a supine position with arms by their side. Number of bed positions: 1–2 (depending on area of interest) × 10 min.

#### Arthroplasty/arthrodesis imaging

Adminstered activity: 8–10 MBq/kg of ^99m^Tc-labelled bisphosphonates (HDP/HMDP, MDP or DPD) injected during a dynamic SPECT acquisition for 90 s while the patient is positioned on the camera. This may be preceded by an auxiliary CT for attenuation correction purposes. Next, a 5-minute blood pool SPECT is acquired 2–7 min after tracer administration. The late-phase acquisition is performed 2–3 h post tracer injection. Patients empty their bladder immediately prior to acquisition if it will be within the field of view. The patient is in a supine position with arms out of the field of view. Number of bed positions: 1 × 8 min over the joint of interest and 1 × 5 min for adjacent joints to assess referred pain syndromes, if desired.

## Future prospects

The currently available full-ring 360° CZT SPECT/CT platforms are ideal stepping stones for further innovation. For example, additional advances are likely to overcome the current limitations in the photon energy window due to the fixed collimator designs. In addition, future SPECT/CT designs will benefit from advances in CT technology, with the adoption of photon-counting detectors, where incident X-ray photon energies are directly recorded as electronic signals. This technology results in ultra-high spatial resolution and lower dose scanning for all body regions, with particular benefit in musculoskeletal CT [47]. In particular, this can be especially useful when performing multi-phase bone SPECT/CT protocols requiring more than one CT acquisition, when software-based registration algorithms may not be effective due to differences in patient positioning between acquisitions. Lastly, the AI revolution will produce the tools necessary to (semi-)automatically analyze and assist in interpreting the extensive and detailed datasets created by these devices, reducing the case workload of nuclear medicine physicians [48].

While beyond the scope of this position paper, it is clear that departments will have to carefully consider their scanner replacement strategy in the coming years based on their study portfolio and referral patterns. Indeed, utilizing a full-ring 360° CZT SPECT/CT system for all types of nuclear medicine studies, such as thyroid, renograms, gastric emptying, hepatobiliary, and sentinel node localization, may require further development from equipment manufacturers and necessitate further adaptations to accommodate these diverse applications. Moreover, to fully understand the clinical benefits and potential limitations of implementing such advanced systems across a wide range of nuclear medicine procedures, it is imperative that more comprehensive clinical studies be conducted. These studies will be essential in providing the necessary evidence to guide best practices and ensure optimal patient outcomes in diverse clinical settings.

## Conclusion

Full-ring 360° CZT technology is an exciting development that gives SPECT/CT near PET/CT resolution and fully leverages the benefit of 3D imaging in an acceptable time envelope of around 20 min for a whole-body scan while potentially reducing radiation exposure to the patient. This technology will likely become mainstream and cause a paradigm shift in nuclear medicine practice, not only for theranostic applications. Now is the time to let go of planar imaging and embrace the full potential of full-ring SPECT/CT, including dynamic and blood pool imaging. In anticipation of a broader evidence base, this position paper will hopefully inspire the use and future research into this exciting technology, including applications beyond bone imaging.

## Data Availability

The present study did not include any specific data analysis. Therefore, no data is available.
